# Investigation of FGF21 mRNA levels and relative mitochondrial DNA copy number levels and their relation in nonalcoholic fatty liver disease: a case-control study

**DOI:** 10.3389/fmolb.2023.1203019

**Published:** 2023-06-06

**Authors:** Massoud Houshmand, Vahide Zeinali, Amirhossein Hosseini, Atena Seifi, Bardia Danaei, Sharareh Kamfar

**Affiliations:** ^1^ Department of Medical Genetics, National Institute for Genetic Engineering and Biotechnology, Tehran, Iran; ^2^ Research Institute for Children’s Health, Shahid Beheshti University of Medical Sciences, Tehran, Iran; ^3^ Pediatric Gastroenterology, Hepatology, and Nutrition Research Center, Research Institute for Children’s Health, Shahid Beheshti University of Medical Sciences, Tehran, Iran; ^4^ Pediatric Nephrology Research Center, Research Institute for Children’s Health, Shahid Beheshti University of Medical Science, Tehran, Iran; ^5^ Department of Microbiology, School of Medicine, Shahid Beheshti University of Medical Sciences, Tehran, Iran; ^6^ Pediatric Congenital Hematologic Disorders Research Center, Research Institute for Children’s Health, Shahid Beheshti University of Medical Sciences, Tehran, Iran

**Keywords:** fibroblast growth factor 21, mitochondrial DNA, nonalcoholic fatty liver disease, FGF21, NAFLD

## Abstract

**Background:** Although the exact mechanisms of nonalcoholic fatty liver disease (NAFLD) are not fully understood, numerous pieces of evidence show that the variations in mitochondrial DNA (mtDNA) level and hepatic Fibroblast growth factor 21 (FGF21) expression may be related to NAFLD susceptibility.

**Objectives:** The main objective of this study was to determine relative levels of mtDNA copy number and hepatic FGF21 expression in a cohort of Iranian NAFLD patients and evaluate the possible relationship.

**Methods:** This study included 27 NAFLD patients (10 with nonalcoholic fatty liver (NAFL) and 17 with non-alcoholic steatohepatitis (NASH)) and ten healthy subjects. Total RNA and genomic DNA were extracted from liver tissue samples, and then mtDNA copy number and FGF21 expression levels were assessed by quantitative real-time PCR.

**Results:** The relative level of hepatic mtDNA copy number was 3.9-fold higher in patients than in controls (*p* < 0.0001). NAFLD patients showed a 2.9-fold increase in hepatic FGF21 expression compared to controls (*p* < 0.013). Results showed that hepatic FGF21 expression was positively correlated with BMI, serum ALT, and AST levels (*p* < 0.05). The level of mitochondrial copy number and hepatic FGF21 expression was not significantly associated with stages of change in hepatic steatosis. Finally, there was a significant correlation between FGF21 expression and mitochondrial copy number in NAFLD patients (*p* = 0.027).

**Conclusion:** Our findings suggest a considerable rise of hepatic FGF21 mRNA levels and mtDNA-CN and show a positive correlation between them in the liver tissue of NAFLD patients.

## Introduction

Chronic liver diseases are rapidly growing as health priorities globally. Fatty liver disease can occur in the setting of both nonalcoholic fatty liver disease (NAFLD) and alcoholic liver disease (ALD) (Toshikuni et al., 2014). NAFLD is among the most prevalent chronic liver disorders worldwide, with a pooled global prevalence of 25.24% and over the past 2 decades there has been a steady increase in its incidence across many populations. (Mitra et al., 2020). NAFLD is characterized by steatosis affecting more than 5% of hepatocytes in individuals who do not consume excessive amounts of alcohol, do not have other liver diseases, and do not take steatogenic drugs. The histological spectrum of NAFLD comprises nonalcoholic fatty liver (NAFL), which involves steatosis without hepatocellular injury, and steatohepatitis (NASH), which involves inflammation and hepatocyte ballooning degeneration in addition to steatosis. Patients with NAFLD can progress to fibrosis, and ultimately, cirrhosis (Chalasani et al., 2012; Younossi et al., 2018). Patients with cirrhosis are at risk of potentially life-threatening liver-related complications such as portal hypertension, hepatic failure, and hepatocellular carcinoma (Ascha et al., 2010; Bhala et al., 2011; Nusrat et al., 2014). Knowledge about the mechanisms that are potentially involved in the pathogenesis of NAFLD is still incomplete. However, new evidence suggests that the pathogenesis of NAFLD involves complex mechanisms collectively referred to as the “multiple parallel hits hypothesis”. This theory proposes that multiple components act in parallel to contribute to the development of NAFLD, rather than in a linear series (Lonardo et al., 2017; Caturano et al., 2021). According to this theory, these factors are believed to play role in the development of NAFLD: insulin resistance (IR), genetic and epigenetic factors, mitochondrial dysfunction, endoplasmic reticulum stress, microbiota, chronic low-grade inflammation, and dysfunction of adipose tissue (Acierno et al., 2020).

The pathogenesis of NAFLD is impacted by changes in the mitochondria, such as mitochondrial DNA depletion, as well as modifications in the beta-oxidation and respiratory chain functions. (Pessayre and Fromenty, 2005). If mitochondrial and peroxisomal functions are unable to handle the increased lipid flow, it can cause respiratory oxidation to collapse, leading to disruption in lipid balance, production of harmful metabolites, and an excess of reactive oxygen species (ROS) (Begriche et al., 2006; Wang et al., 2020). These events contribute to oxidative stress, hepatic necro-inflammatory processes, and worsening of mitochondrial damage. In fact, it has been proven that mitochondrial dysfunction is directly associated with IR, obesity, and the release of pro-inflammatory cytokines levels like tumor necrosis factor-alpha (TNF-α) (Paradies et al., 2014). Furthermore, ROS and oxidized low-density lipoprotein (LDL) cholesterol particles can activate Kupffer and hepatic stellate cells, leading to the deposition of collagen and the progression of liver fibrosis (Cusi, 2009).

In addition, endoplasmic reticulum (ER) malfunction probably leads to accumulation of unfolded proteins inside the ER, increased protein synthesis, reduction of Adenosine triphosphate (ATP), and activation of the unfolded protein response (UPR). UPR is a compensatory response to decrease protein synthesis, increase protein trafficking capacity through the ER, and increase protein degradative pathways (Wang and Kaufman, 2014). UPR failure to solve the protein-folding defect, may induce hepatocytes apoptosis.

Based on the mentioned multi-hit hypothesis, and factors like ATP deficiency, increased lipid flow, and dysfunction of beta-oxidation which are directly linked to mitochondria, several studies have suggested that NAFLD might be a mitochondrial disease (Begriche et al., 2006; Dornas and Schuppan, 2020; Xu et al., 2021). This condition leads to mitochondrial damage and mitochondrial DNA copy number (mtDNA-CN) variations in hepatocytes (Pirola et al., 2015; Kamfar et al., 2016).

Circulating fibroblast growth factor 21 (FGF21), a member of the FGF family, is predominantly liver-derived and is involved in the hormonal regulation of glucose and lipid metabolism, energy homeostasis, insulin sensitivity, and other metabolic functions (Cuevas-Ramos et al., 2009). Numerous preclinical and clinical evidence suggested that aberrant FGF21 signaling may play a role in the pathogenesis and progression of NAFLD (Liu et al., 2015; Rusli et al., 2016; Tucker et al., 2019). Both FGF21 serum levels and FGF21 expression were discussed to be indicators of NAFLD (Falamarzi et al., 2022). It has been shown that FGF21 stimulates lipolysis by decreasing fat stores leading to reducing hepatic steatosis and lipotoxicity (Xu et al., 2009; Tanaka et al., 2015; Bao et al., 2018). Moreover, several studies have also reported that FGF21 reduces oxidative stress and endoplasmic reticulum stress (Ye et al., 2014; Boparai et al., 2015) and enhances mitochondrial function (Lee et al., 2016).

In this observational case-control study, considering the role of FGF21 and mitochondria in NAFLD pathogenesis, our first aim was to assess FGF21 expression and mtDNA-CN in Iranian NAFLD patients in different stages of the disease and compare those to healthy control group. Our second objective was to investigate the possible relation between the levels of FGF21 expression and mtDNA-CN in liver samples from Iranian NAFLD patients.

## Methods

### Study population

Over one and a half years, this study was carried out on NAFLD patients identified from a sub-specialist tertiary NAFLD clinic at the Khatam Ol-Anbia Hospital, Tehran, Iran. Participants were selected based on liver ultrasonography, clinical, and laboratory findings. Patients with other liver problems, cancer, family history of diabetes, history of alcohol drinking, viral hepatitis (B or C), steatogenic medications, and glucocorticoid therapy were carefully excluded. Control group consisted of healthy volunteers who did not have any history of liver and metabolic related disorders. A liver needle biopsy was used to obtain liver samples. two to three samples were obtained from each participant which were used for histological assessment and nucleic acid extraction. Relevant clinical and laboratory data were collected from the time of liver biopsy. Patients with NAFLD were classified into NAFL and NASH based on the histologic findings and were approved by at least two pathologists. The same method was used for control group classification and individuals with pathologic findings in their samples were excluded from the control group. The medical ethics committee approved the Protocol of Hamadan University of Medical Sciences (P/16/35/9/3481), and all the participants signed Written Informed Consent before participating in this research study. The study protocol conformed to the ethical guidelines of the World Medical Association Declaration of Helsinki.

### Anthropometric assessment and results of biochemical analysis

Relevant clinical details such as age, gender, weight, and height were obtained from all patients at the time of liver biopsy. The body mass index (BMI) was calculated by the formula: weight (kg)/height^2^ (m^2^). Blood tests taken at the time of liver biopsy were used to determine related paraclinical parameters. An automated enzymatic procedure assayed with alanine transaminase (ALT), aspartate transaminase (AST), cholesterol, triglyceride, HDL cholesterol, and fasting blood glucose (FBS) levels. The Friedewald formula was used for calculation of LDL-cholesterol levels.

### Histological assessment

Percutaneous liver biopsies were performed using a Menghini needle. Liver biopsies were all >15 mm in length and were read by two experienced hepatopathologists. And any disagreement was resolved with discussion between them. “NASH” was defined as steatosis with hepatocyte ballooning degeneration and inflammation with or without fibrosis (Yeh and Brunt, 2014). “NAFL” was defined as steatosis only, or steatosis with mild inflammation without hepatocyte ballooning degeneration.

### DNA/RNA extraction

Genomic DNA and total RNA were extracted simultaneously from fresh liver samples using the AllPrep DNA/RNA Micro (Qiagen, Dubai, United Arab Emirates). According to the manufacturer’s instructions, hepatic tissue samples were first lysed and homogenized in a buffer for inhibition of DNases and RNases to obtain intact DNA and RNA. The lysate was passed through an AllPrep DNA spin column to selectively and efficiently isolate DNA. The column was then washed and DNA was eluted. Ethanol was added to the flow-through from the AllPrep DNA spin column to allow proper binding conditions for RNA, and the sample was then applied to RNeasy MinElute spin column, where total RNA binds into the membrane and contaminants were effectively washed away. Finally, RNA was then eluted in water.

### Mitochondrial DNA copy number

Quantification of mtDNA-CN was assessed using quantitative real-time PCR (qPCR). According to the manufacturer’s Protocol, this assay was carried out using the SYBR master mix (Real qPCR 2x Mix, Amplicon, Wrocław, Poland). Amplification was done with two pair primers: ONP86/ONP89 and B-actin (Shakhssalim et al., 2013; Kamfar et al., 2016; Zabihi Diba et al., 2016). The first set of primers (86/89) was used to amplify a normal fragment in mtDNA, and the second set (B-actin) was used as an internal control for nucleic DNA. Primer-BLAST was used to check the primer specificity (Ye et al., 2012). The amplification was done for 40 cycles using the following conditions: 95°C for 15 min, then 95°C for 30 s, and 58°C for 1 min. All samples were run in triplicate. Relative levels of mtDNA-CN were measured by using the 2^−ΔΔCT^ method (Jensen, 2012).

### Hepatic mRNA expression of FGF21

RNA isolated from liver biopsy was reverse transcripted using the cDNA Synthesis Kit (Thermo Fisher Scientific, Waltham, MA, United States of America) and the obtained cDNA was used as template in the qPCR reaction. According to the manufacturer’s Protocol, the quantitative real-time PCR assay was carried out using specific primers in a 20 µL reaction volume containing SYBR Green master mix (Real QPCR 2x Mix, Amplicon, Wrocław, Poland). Each reaction was run in duplicate, and the accuracy of qPCR product size was confirmed by gel electrophoresis. Two primer pairs were designed to analyze FGF21 and B-actin as housekeeping genes (Table 1).

**TABLE 1:** Sequences of primers used for quantitative real-time PCR.

*B-actin*	Forward	5′- AGA​CGC​AGG​ATG​GCA​TGG​G-3′	161bp	P60709	Accession number
	Reverse	5′- GAG​ACC​TTC​AAC​ACC​CCA​GCC-3			
*FGF21*	Forward	5′-TCA​AGA​CAT​CCA​GGT​TCC-3′	109 bp	Q9NSA1	
	Reverse	5′-TAT​CCG​TCC​TCA​AGA​AGC-3′			

### Statistical analysis

Analyses were performed using SPSS 18.0 software package (SPSS Inc., United States). Data were presented as means ± standard deviation (SD). The student's t-test was used for comparing normally distributed variables. Logistic regression was used to adjust for age, BMI, and lipid level confounders. Pearson correlation coefficient was carried out to describe the relationship of FGF21 expression and mtDNA-CN with variables related to NAFLD. In all statistical tests, *p* < 0.05 was regarded as statistically significant.

## Results

**FIGURE 1:**
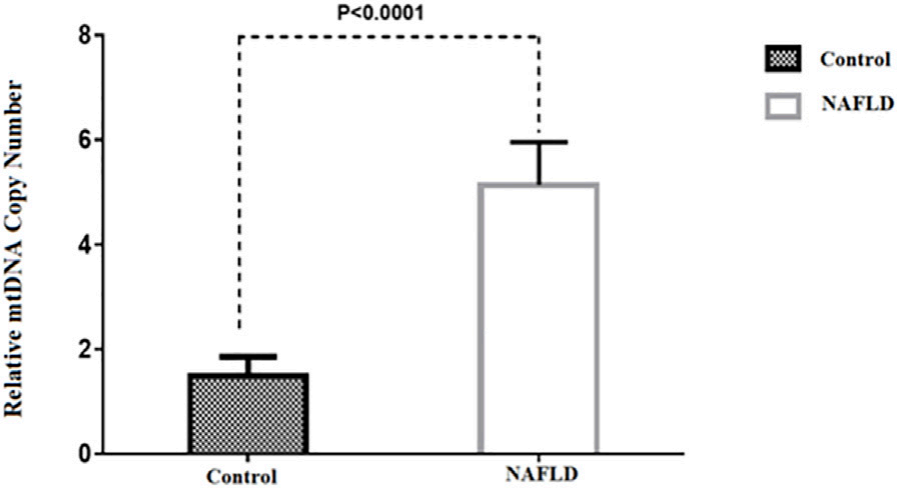
Comparison of the relative mtDNA copy number between NAFLD and control group. *p-value was computed *t*-test and data are shown as Mean ± SEM.

Baseline characteristics of participants, including 27 patients with NAFLD (17 NASH, 10 NAFL) and ten healthy control subjects, are presented in Table2. The mean TG, ALT, AST, and BMI in NAFLD patients were significantly higher than in the control group (*p* < 0.05). No statistically significant difference was found in the other parameters between these two groups. According to the results, we observed a 3.9-fold increase in relative mtDNA-CN in the livers of NAFLD patients compared to healthy controls (*p* < 0.0001) (Figure1). A comparison of mtDNA-CN showed a 4.3 (*p* < 0.008) and 3.5-fold (*p* < 0.013) increase in patients with NAFL and NASH compared to control subjects, respectively. No substantial differences were observed in mtDNA-CN between the NAFL and NASH patients (*p* < 0.615) (Supplementary Table S1). No relation was observed after adjustment for age and BMI between mtDNA-CN and variables such as lipid levels and blood pressure (*p* > 0.05). In addition, no significant association was found between mtDNA-CN and age (*p* > 0.05) (Supplementary Table S2).

**TABLE 2:** Anthropometrics parameters and biochemical indexes among control and NAFLD group.

Parameter	NAFLD (*n* = 27)	Control (*n* = 10)	P value^α^
Gender (Male/Female)	(11/16)	(2/8)	0.440
Age (year)	43.19 ± 9.60	38.00 ± 8.96	0.143
Body Mass Index (kg/m2)	44.21 ± 9.90	26.90 ± 2.56	<0.001^a^
Systolic blood pressure (mmHg)	120.37 ± 10.37	115.0 ± 7.07	0.141
Diastolic blood pressure (mmHg)	75.07 ± 5.99	74.00 ± 5.16	0.620
LDL-Cholesterol (mmol/L)	100.14 ± 32.59	110.50 ± 24.36	0.381
HDL-Cholesterol (mmol/L)	46.04 ± 9.70	54.80 ± 6.07	0.012
Triglycerides (mmol/L)	193.0 ± 80.27	129.8 ± 36.13	0.023^a^
Total Cholesterol (mmol/L)	187.85 ± 29.87	188.3 ± 28.04	0.967
FBS (mmol/L)	112.30 ± 28.94	115.1 ± 34.72	0.806
ALT (U/L)	38.65 ± 23.72	16.00 ± 3.80	<0.001^a^
AST (U/L)	25.68 ± 12.24	17.30 ± 1.57	0.002^a^
ALP (U/L)	189.74 ± 65.38	175.50 ± 34.86	0.519

*p* values were computed by t-test.

Abbreviations: FBS: fasting blood glucose; HDL: high-density lipoprotein; LDL: low-density lipoprotein; ALT: Alanine transaminase, AST: Aspartate Aminotransferase, ALP: Alkaline Phosphatase Test.

^a^ Statistically significant.

^b^ Values are presented as mean±SD, or median (interquartile range).

**FIGURE 2:**
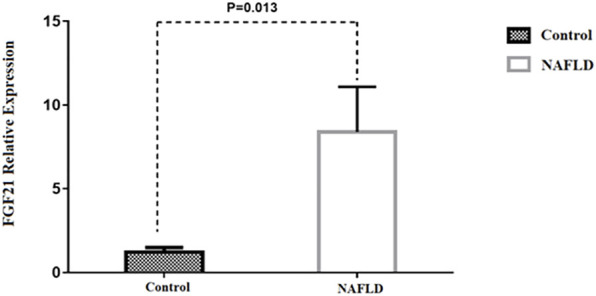
Comparison of the FGF21 relative expression between NAFLD and control group. *p-value was computed by *t*-test and data are shown as Mean ± SEM.

Our findings also indicated that FGF21 expression in hepatic tissue was 2.9-fold higher in patients with NAFLD than in control subjects (*p* = 0.013) (Figure 2). No significant difference was observed between patients with NAFL and NASH (*p* = 0.843) (Supplementary Table S1). Our results also showed a positive correlation between FGF21 expression and BMI (*p* = 0.035), AST (*p* = 0.02), and ALT (*p* < 0.01) levels (Supplementary Table S2). We found no sex difference in the expression of FGF21 between NAFL and NASH groups (*p* > 0.05). In addition, there was no significant relation between FGF21 expression and other anthropometric and biochemical measurements after adjusting for age and BMI in these groups.

**FIGURE 3:**
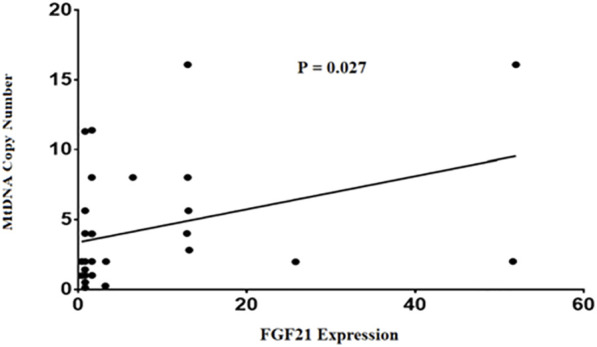
Comparison of the relative mtDNA copy number and FGF21 expression in NAFLD patients.

Finally, our findings in this study demonstrated a positive correlation between mtDNA-CN and FGF21expression levels in NAFLD patients (*p* = 0.027) (Figure 3). This comparison remained statistically significant after adjusting for age and BMI (*p* = 0.035).

## Discussion

Recent studies have shown that NAFLD can be classified as a mitochondrial disease, in fact mitochondrial malfunction leads to abnormal hepatic fatty acid oxidation causing fat accumulation and hepatic steatosis. In addition to that, such mitochondria produce more ROS and less ATPs which cause more damages to mitochondria and hepatic cells making a destructive cycle (Koroglu et al., 2016; Dornas and Schuppan, 2020; Dabravolski et al., 2021). Thus, measuring relative mtDNA-CN as determining factor for mitochondrial damage and activity rate can be helpful for identifying the stage and prognosis of the disease (Cao et al., 2020; Filograna et al., 2021). On the other hand, in some stages of different mitochondrial disorders mtDNA-CN can be within the normal range and its changes should be interpreted along with clinical, histological, and other laboratory findings (Mavraki et al., 2023). However, despite its flows, mtDNA-CN is widely accepted among researchers as a useful method to assess mitochondrial function (Castellani et al., 2020; Zhang et al., 2022). Our results showed significant elevation in relative mtDNA-CN in NAFLD patients’ liver which were in accordance with our previous work (Kamfar et al., 2016). Also, Malik et al. demonstrated a rise in hepatic mtDNA content during the initial stages of hepatic steatosis in mice models (Malik et al., 2019). Chiappini et al. also found that the mtDNA to nuclear DNA (nDNA) ratio was higher in hepatic steatosis than in normal liver tissues (Chiappini et al., 2006). However, in contrast to these results Sookian et al. and Pirola et al. reported significant lower mtDNA/nDNA ratio in the liver of NAFLD patients compared to that of healthy control group (Sookoian et al., 2010; Pirola et al., 2015). Studies suggested that mtDNA-CN upregulation in humans with mitochondrial diseases, mostly concomitant with an overall rise in mitochondrial biogenesis, is regularly occurring and typically considered as a compensatory mechanism to support cellular bioenergetics (Hsin-Chen et al., 2000; Lee et al., 2018; Skuratovskaia et al., 2019; Filograna et al., 2021). We assumed that in NAFL patients this compensatory mechanism leads to mtDNA-CN upregulation and when this mechanism fails, due to disease progression and significant higher oxidative stress in NASH patients, the mtDNA-CN falls down. On the other hand, an increase in defective mitochondria can be considered detrimental rather than protective because of ROS accumulation as byproducts of the defective mitochondria (Sanyal et al., 2001; Shami et al., 2021). So, the precise involvement of mitochondrial biogenesis in these patients remains a topic of debate. In the present study we investigate NAFL and NASH patients separately in this regard. Although in our results relative mtDNA-CN was higher in NASH samples compared to NAFL ones, the difference was not significant. Thus, the interpretation of this data can be challenging, partly due to method‐, specimen‐ and study design‐related issues.

Some studies reported substantial depletion of mtDNA-CN in hepatic cells with aging in animal models (Barazzoni et al., 2000; Hartmann et al., 2011). Wachsmuth et al. suggested that mtDNA-CN decreased with age in human muscle tissue (Wachsmuth et al., 2016). However, Frahm et al. reported no age-related increase of mtDNA amount in brain, skeletal muscle and human heart (Frahm et al., 2005). Their result was in accordance with our present and previous studies (Kamfar et al., 2016) on human liver cells. This can have several reasons, for example, beside limited number of samples, our studies had a case-control design and we did not investigate liver mtDNA-CN in individuals through long duration of time. Also, evidence indicated that mtDNA-CN can vary between different cell types and answer differently to various physiologic and pathologic states including NAFLD (Tapia et al., 2018; Ma et al., 2020; Filograna et al., 2021) suggesting a dynamic nature for this parameter.

In the current study, we detected a significant increase in FGF21 expression in patients with higher BMI. This results were in concordance to many previous studies conducted on children (Reinehr et al., 2012) and adults (Dushay et al., 2010; Tyynismaa et al., 2011). According to our results, FGF21 expression rise was also significantly correlated to high AST and high ALT. Nakanishi et al. supported this result in their study which showed that FGF21 level was remarkably associated with AST and ALT elevation (Nakanishi et al., 2021). This emphasizes FGF21 role as an ameliorating agent which its production increases in liver injuries.

Numerous evidence showed that circulatory FGF21 rise in NAFLD patients and discussed its role as a protective factor (Tucker et al., 2019; Tillman and Rolph, 2020). FGF21 elevation can be seen as another compensatory mechanism in these patients. However, we do not fully understand the molecular regulatory mechanisms behind its function yet (Watanabe et al., 2020; Tan et al., 2023). In our study we detected remarkable increase in its expression in liver tissue of NAFLD patients compared to of control group. This result was supported by other previous works (Kamfar et al., 2016; Liu et al., 2023). Li et al. suggested that FGF21 may mirror the severity and progression of NAFLD due to its association with obesity, triglyceride, and gama-glutamyltransferase (Li et al., 2010). They reported that hepatic FGF21 mRNA expression in NAFLD patients with grade 1 was 4-fold higher than that in grade 0 (*p* < 0.01), and grade 2–3 was 14.71-fold higher than that in grade 0 (*p* < 0.01). In another study conducted by Flisiak-Jackiewicz et al. serum FGF21 levels were higher significantly in obese children with NAFLD compared to obese children without the disease and had a positive correlation with steatosis grades in biopsies (Flisiak-Jackiewicz et al., 2019). However, we did not detect any significant difference in its expression between NAFL and NASH patients. This can be due to the failure of FGF21 related compensatory mechanisms in more advanced stages of the disease. In this regard Alisi et al. found that FGF21 levels increased progressively with the increase of hepatic steatosis, but when hepatic fat content reached the fourth quartile, FGF21 levels tended to decline (Alisi et al., 2013). The authors of that study suggested that decreased production of this molecule by hepatocytes due to their injury or death caused by lipotoxicity and hepatic inflammation may be the cause of its decline in adult patients with severe liver steatohepatitis. Their results were also in accordance with the study conducted by Dushay et al. who reported lower hepatic FGF21 expression in NASH compared to NAFLD and suggested it may reflect more advanced hepatic injury (Dushay et al., 2010).

Finally, we reported a positive correlation between mtDNA-CN and FGF21 expression levels in liver tissue samples of patients with NAFLD which as mentioned before might be a mitochondrial disease. Based on current results, these two factors may play critical roles at the early stages of disease in inhibiting NAFLD development to NASH. In a recent systematic review, Lin et al. reported that FGF21 is highly sensitive and specific for diagnosis of mitochondrial diseases (Lin et al., 2020). According to a study conducted by Ji et al. FGF21 expression in mitochondrial diseases increases as a compensatory mechanism in energy metabolism. Furthermore, they showed that FGF21 regulates energy homeostasis by increasing expression of mitochondrial genes and mtDNA-CN (Ji et al., 2015). A more accurate explanation of the relation between these two factors in NAFLD remains to be investigated. Despite the importance of this matter, there are currently no approved therapies for treating NAFLD or NASH globally (Francque and Vonghia, 2019). Exercise prescription is considered a central strategy in treatment (van der Windt et al., 2018). In confirmation of this proposed option, in one study, J Henkel et al. showed that exercise improved glucose tolerance in NAFLD by inducing FGF21 production by the liver (Henkel et al., 2019).

Our study holds some limitations. Firstly, limited number of participants is an important barrier for making a reliable conclusion that needed to be noticed. Secondly, because of the dangers of sample collection in this study we could not match our controls with patients perfectly. We tried to minimize the effect of this bias by adjusting for some confounding factors like age and BMI in our analysis. Thirdly, we did not collect data during the progression of the disease in individuals hence our results regarding changes in parameters during disease progression are subject to error. Finally, like any study using liver biopsy as a standard, miss-diagnosis of disease stage at biopsy can be caused by sampling error (Ratziu et al., 2005). The potential for sampling error in this study was minimized by collecting 2 to 3 biopsies >15 mm from each participant and consulting two hepatopathologists to examine each sample.

In the present study we did not collect data on FGF21 protein levels in liver tissues and blood samples of patients and just reported and analyzed FGF21 mRNA levels. It has been shown that the protein levels can be independent of associated mRNA levels in tissues and blood (Greenbaum et al., 2003; Silva and Vogel, 2016). This can be caused by several factors affecting FGF21 production like mRNA degradation, translation, and protein degradation (Battle et al., 2015; Bayoumi et al., 2021). Hence, making it challenging to find a direct cause-and-effect relationship between different gene expressions. We tried to tackle this issue by measuring mRNA levels which become affected prior to changes in protein levels and to help clarifying ambiguities. More studies with larger number of participants and measuring protein levels through disease progression are needed to track the changes more specifically.

As for studying mitochondrial changes we only collected data on mtDNA-CN in liver tissues of participants as previous literature suggested it to be a biomarker of mitochondrial function (Castellani et al., 2020; Zhang et al., 2022). However, further studies on mitochondrial changes with different methods like electron microscopy and evaluation of consequences of mtDNA-CN changes are needed.

Taken together, in this case-control study, we have shown a considerable rise in FGF21 expression and mtDNA-CN in NAFLD patients compared to healthy control group. While the data presented here suggest that mitochondrial dysfunction and FGF21 expression are involved in the disease mechanism, they are not conclusive in predicting prognosis or progression of the disease. Furthermore, our results suggested a positive correlation between hepatic FGF21 expression and mtDNA-CN in the liver tissue of NAFLD patients. Further research is needed to determine the exact relationship between mtDNA-CN and FGF21 with NAFLD susceptibility.

## Data Availability

The original contributions presented in the study are included in the article/Supplementary Materials, further inquiries can be directed to the corresponding author.

## Supplementary material

SUPPLEMENTARY TABLE S1 Comparison of mtDNA-CN between NAFL and NASH patients and controls.

SUPPLEMENTARY TABLE S2 Correlations of serum fgf21 expression with copy number anthropometric parameters and biochemical indexes.
