# Supplementary material for: Investigation of FGF21 mRNA levels and relative mitochondrial DNA copy number levels and their relation in nonalcoholic fatty liver disease: a case-control study
Source: Front Mol Biosci. 2023 Jun 6;10:1203019. doi: 10.3389/fmolb.2023.1203019 (PMC10279952; doi:10.3389/fmolb.2023.1203019)
Supplement: Supplementary file 1 [file Table1.docx]

**Supplement table 1: Comparison of mtDNA-CN between NAFL and NASH patients and controls**

|  | Control | NAFL | NASH | P-value^*^ | P-value^**^ | P-value^***^ |
| --- | --- | --- | --- | --- | --- | --- |
| Exp.86-89 | 1.48±1.16 | 5.68±4.29 | 4.80±4.35 | 0.013 | 0.008 | 0.615 |
| Exp.fgf21 | 1.21±0.85 | 9.14±15.68 | 7.95±13.32 | 0.145 | 0.054 | 0.843 |

P-value^*^=the relationship between control and NAFL, P-value^**^= the relationship between control and NASH, P-value^***^= the relationship between NAFL and NASH.
