# Supplementary material for: Investigation of FGF21 mRNA levels and relative mitochondrial DNA copy number levels and their relation in nonalcoholic fatty liver disease: a case-control study
Source: Front Mol Biosci. 2023 Jun 6;10:1203019. doi: 10.3389/fmolb.2023.1203019 (PMC10279952; doi:10.3389/fmolb.2023.1203019)
Supplement: Supplementary file 2 [file Table2.docx]

| **Supplement table 2: Correlations of serum fgf21 expression with copy number anthropometric parameters and biochemical indexes** | | | | | | | | |
| --- | --- | --- | --- | --- | --- | --- | --- | --- |
| Copy number (age and BMI adjusted) | | Copy number | | FGF21 (age and BMI adjusted) | | fgf21 expression | | Variables |
| P value | r | P value | r | P value | r | P value | r |  |
| 0.035 | 0.358 | 0.027 | 0.363^*^ | - | - | - | - | fgf21 expression |
| 0.011 | 0.426^*^ | 0.009 | 0.421^*^ | 0.035 | 0.358^*^ | 0.156 | 0.238 | BMI |
| - | - | 0.779 | 0.048 | 0.233 | 0.207 | 0.198 | 0.216 | Age |
| 0.190 | -0.227 | 0.148 | -0.243 | 0.910 | 0.020 | 0.915 | 0.018 | HDL |
| 0.328 | -0.170 | 0.218 | -0.207 | 0.467 | -0.127 | 0.289 | 0.179 | LDL |
| 0.669 | 0.075 | 0.670 | 0.072 | 0.238 | 0.205 | 0.168 | 0.232 | TG |
| 0.303 | 0.179 | 0.489 | 0.117 | 0.195 | 0.224 | 0.301 | 0.175 | Chol |
| 0.338 | -0.167 | 0.467 | -0.123 | 0.359 | 0.160 | 0.203 | 0.214 | Systolic Blood Pressure |
| 0.427 | -0.139 | 0.534 | -0.105 | 0.187 | 0.229 | 0.113 | 0.265 | Diastolic Blood Pressure |
| 0.433 | 0.137 | 0.377 | 0.150 | 0.356 | 0.161 | 0.224 | 0.205 | FBS |
| 0.172 | 0.236 | 0.103 | 0.272 | 0.010 | 0.428^*^ | 0.015 | 0.398^*^ | ALT |
| 0.252 | 0.199 | 0.196 | 0.217 | 0.023 | 0.382^*^ | 0.013 | 0.406^*^ | AST |
| 0.614 | -0.088 | 0.639 | -0.080 | 0.660 | 0.077 | 0.476 | 0.121 | ALP |

* Correlation is significant at the 0.05 level
